# Molecular and Regenerative Effects of Platelet-Rich Plasma and Related Hemocomponents in Animal Models of Liver Injury—A Systematic Review

**DOI:** 10.3390/ijms27021013

**Published:** 2026-01-20

**Authors:** Jorge U. Carmona, Julián David Hernández-Valencia, Catalina López

**Affiliations:** 1 Grupo de Investigación Terapia Regenerativa, Departamento de Salud Animal, Universidad de Caldas, Calle 65 No 26-10, Manizales 170004, Colombia; julian.hernandez57287@ucaldas.edu.co; 2 Grupo de Investigación Patología Clínica Veterinaria, Departamento de Salud Animal, Universidad de Caldas, Calle 65 No 26-10, Manizales 170004, Colombia; catalina.lopez@ucaldas.edu.co

**Keywords:** platelet-rich plasma, liver regeneration, hepatoprotection, fibrosis, oxidative stress, growth factors, molecular therapy, cytokines

## Abstract

Platelet-rich plasma (PRP) has been increasingly explored as a biologic strategy for liver repair; however, preclinical studies have evaluated not only intact PRP but also PRP related hemocomponents with distinct biological properties, complicating interpretation and translation of the evidence. A systematic review of experimental studies was conducted to assess the effects of PRP and related hemocomponents in animal models of liver injury, focusing on molecular, metabolic, biochemical, and histological outcomes, and evaluating methodological quality and risk of bias using the Cochrane ROB 2.0 framework. Fourteen eligible studies were identified across toxic, cholestatic, parasitic, radiation-induced, and surgical models. Platelet-based interventions were generally associated with hepatoprotective, antifibrotic, antioxidant, immunomodulatory, and pro-regenerative effects; however, responses were highly context dependent and varied according to injury etiology, disease stage, administration route and timing, and the frequent use of combination therapies. Substantial heterogeneity in the platelet-based products evaluated—including platelet supernatants and lysates—and inconsistent reporting of key compositional parameters limited product classification, cross-study comparability, and mechanistic interpretation, while ROB 2.0 assessments revealed predominantly some concerns of bias. PRP and related hemocomponents show biologically relevant effects in experimental liver injury, but their translational potential is constrained by methodological heterogeneity and inadequate product characterization. Standardized reporting, controlled comparative designs, and clinically relevant models are required to clarify efficacy and support rational translation.

## 1. Introduction

Liver regeneration represents one of the most complex processes of tissue repair, involving a coordinated balance between inflammation, oxidative stress, angiogenesis, and cellular proliferation [1,2,3]. Despite the intrinsic regenerative capacity of hepatocytes, the progression of acute or chronic liver injury often leads to irreversible fibrosis and organ dysfunction [4,5]. Current therapeutic approaches for hepatic repair remain limited, and most are unable to fully restore the structural and functional integrity of the liver [6,7,8]. Hence, biological therapies that stimulate endogenous regeneration and modulate molecular repair pathways have gained increasing attention [9,10,11,12].

Platelet-rich plasma (PRP) has emerged as a promising autologous or allogeneic biotherapy within regenerative medicine [13,14,15,16]. Obtained from centrifuged, anticoagulated whole blood, PRP contains a high concentration of platelets that release a wide range of bioactive molecules, including vascular endothelial growth factor (VEGF), hepatocyte growth factor (HGF), transforming growth factor-beta 1 (TGF-β1), platelet-derived growth factor (PDGF), epidermal growth factor (EGF), and insulin-like growth factor type 1 (IGF-1) [17,18]. These molecules regulate essential cellular functions such as proliferation, angiogenesis, extracellular matrix remodeling, and inflammation control, thereby creating a favorable microenvironment for tissue regeneration [13,14,15,16].

Experimental studies indicate that PRP can activate cytoprotective and antifibrotic pathways—including nuclear factor erythroid 2–related factor 2 (Nrf2)-regulated antioxidant responses, vascular-endothelial growth factor (VEGF)- and hepatocyte growth factor (HGF)-mediated regenerative signaling—while downregulating key profibrotic and pro-apoptotic mediators such as TGF-β1, alfa–smooth muscle actin (α-SMA), Caspase-3, and nuclear factor kappa B (NF-κB). These molecular effects have been described across different models of toxic, metabolic, or fibrotic liver injury. However, the magnitude and consistency of these responses vary considerably among studies, largely due to methodological heterogeneity in PRP preparation protocols, platelet and leukocyte concentrations, activation procedures, and administration routes [9]. Importantly, experimental studies have used not only intact PRP but also platelet-derived supernatants, releasates, lysates, and washed platelet preparations, which differ substantially in cellular composition, biological activity, and mechanistic interpretation.

In recent years, several experimental studies have investigated the use of PRP and related hemocomponents, such as platelet lysates and platelet-rich gel supernatants, in animal models of liver injury induced by toxic, surgical, ischemic, or infectious mechanisms. These models have provided valuable insights into the regenerative and hepatoprotective potential of PRP and its interaction with key molecular pathways involved in liver repair. However, despite generally encouraging findings, the available preclinical evidence remains fragmented, and no systematic synthesis has comprehensively evaluated the consistency, methodological quality, and risk of bias of these studies.

Therefore, the present study systematically reviews the experimental evidence on PRP use in animal models of liver injury, with a specific focus on the molecular, metabolic, histological, and biochemical pathways underlying its hepatoprotective and regenerative effects. In addition to summarizing therapeutic outcomes, this review critically assesses methodological quality and risk of bias using the Cochrane ROB 2.0 framework [19]. By integrating mechanistic insights—including antioxidant, antifibrotic, immunomodulatory, and growth factor–mediated pathways—this systematic review aims to identify common trends, sources of heterogeneity, and translational opportunities for optimizing PRP as a biologic therapy in hepatic regeneration.

## 2. Methods

This systematic review was conducted in accordance with the PRISMA 2020 statement and followed the principles of the Cochrane Handbook for Systematic Reviews of Interventions [20,21].

### 2.1. Search Strategy

A comprehensive electronic search was performed across three major databases: PubMed, Scopus, and Web of Science, covering studies published from 1 January 2000 to 30 June 2025. The search was designed to capture all experimental investigations exploring PRP in hepatic injury models, without language restrictions.

The following search terms were applied using Boolean operators:

(“platelet rich plasma” OR “platelet concentrate” OR “PRP”) AND (“hepatic injury” OR “liver disease” OR “hepatitis” OR “liver fibrosis” OR “liver regeneration”) AND (“animal model” OR “experimental model” OR “preclinical study”).

Additional records were retrieved by manually screening the references of relevant articles to ensure comprehensive coverage of the literature.

### 2.2. Eligibility Criteria

The inclusion and exclusion criteria were established according to the PICO framework [22,23]: (1) Population (P): Experimental or animal models with natural or induced liver injury. (2) Intervention (I): Administration of platelet-rich plasma (PRP), regardless of the preparation method, dosage, or route of administration. (3) Comparison (C): Control groups receiving placebo, no treatment, or alternative regenerative interventions. (4) Outcomes (O): Evidence of hepatic regeneration assessed by biochemical (ALT, AST, ALP, GGT, etc.), histological, molecular, or functional parameters.

Studies were included if they: (1) Were original experimental investigations. (2) Used PRP as a therapeutic intervention for liver injury and, (3) reported measurable outcomes related to hepatic repair, fibrosis, or molecular response.

The following types of studies were excluded: (1) Reviews, meta-analyses, case reports, or editorials. (2) In vitro studies or studies not involving liver models and, (3) Reports using platelet-derived extracellular vesicles (PRP-EV) as the sole intervention, due to methodological incompatibility with PRP-based protocols.

### 2.3. Study Selection Process

All records identified were uploaded into Rayyan (Rayyan Systems Inc., Doha, Qatar; accessed 13 October 2025), a free web-based tool designed to support researchers in managing the screening process of systematic reviews [24]. Two reviewers (J.D.H.-V. and C.L.) independently screened titles, abstracts, and keywords to determine relevance. Full-text versions of potentially eligible studies were assessed for inclusion according to the pre-established criteria.

Discrepancies between reviewers were resolved by discussion until consensus was reached with J.U.C. A PRISMA flow diagram was constructed to illustrate the selection process, including identification, screening, eligibility, and inclusion phases [21]. The diagram was generated using the PRISMA2020 R package (Version 1.1.1) and its associated Shiny web application [25].

### 2.4. Data Extraction

A structured data extraction form was developed to ensure consistent collection of relevant information. For each included study, the following data were recorded: (1) Authors, year of publication, and country of origin. (2) Animal species, (3) sample size, and (4) model of hepatic injury (chemical, surgical, infectious, or radiation-induced).

### 2.5. Characterization and Reporting Quality of Platelet-Rich Plasma in Experimental Liver Studies

To overcome the lack of standardization in PRP preparation and reporting across the included studies, we adapted a recently developed methodological framework derived from a previous scoping review [26]. This framework shifts from numerical scoring systems toward minimum reporting standards, consistent with current trends in translational and veterinary regenerative research.

The proposed checklist restructures key methodological domains into seven essential categories, each containing mandatory and recommended reporting items. These elements were adapted from human PRP guidelines [27,28] but tailored to the specific needs of experimental liver injury models, where the biological variability of PRP products has a direct impact on hepatoprotective outcomes.

The seven domains include: (1) blood collection and pre-processing (anticoagulant, blood volume, timing, temperature), (2) PRP preparation (centrifugation parameters, manual vs. commercial protocols), (3) PRP harvesting (layer harvested, final volume), (4) cellular characterization (platelets, leukocytes, erythrocytes; enrichment and yield), (5) biochemical characterization (growth factors, cytokines, activation or lysis method), (6) protein enrichment controls (PPP/plasma controls; positive lysis control), and (7) in vivo administration protocol (dose, route, activation status, rationale, adverse events). A complete version of the adapted checklist for experimental hepatic studies is provided in Table 1, and was used to evaluate completeness and transparency of reporting across studies.

Rather than applying an arbitrary numeric score, each item was assessed as reported, partially reported, or not reported, allowing identification of methodological gaps and reducing bias derived from heterogeneous reporting practices.

This updated framework ensures that PRP characterization in liver regeneration research is evaluated with the same rigor expected in musculoskeletal and clinical regenerative applications, while remaining flexible enough to accommodate diverse experimental designs.

### 2.6. Assessment of Risk of Bias

The methodological quality of the included studies was assessed using the Cochrane Risk of Bias tool (ROB2), adapted for preclinical experiments [29]. This tool evaluates five key domains: (1) bias arising from the randomization process; (2) bias due to deviations from intended interventions; (3) bias due to missing outcome data; (4) bias in outcome measurement; and (5) bias in selection of the reported results. Each domain was rated as low risk, some concerns, or high risk of bias.

To visualize the results, the ROBVIS tool (https://mcguinlu.shinyapps.io/robvis/ (accessed on 10 December 2025)) was used to generate traffic light plots and summary plots, providing an intuitive representation of the overall quality of the evidence base [19].

## 3. Results and Discussion

### 3.1. Study Selection

**Figure 1:**
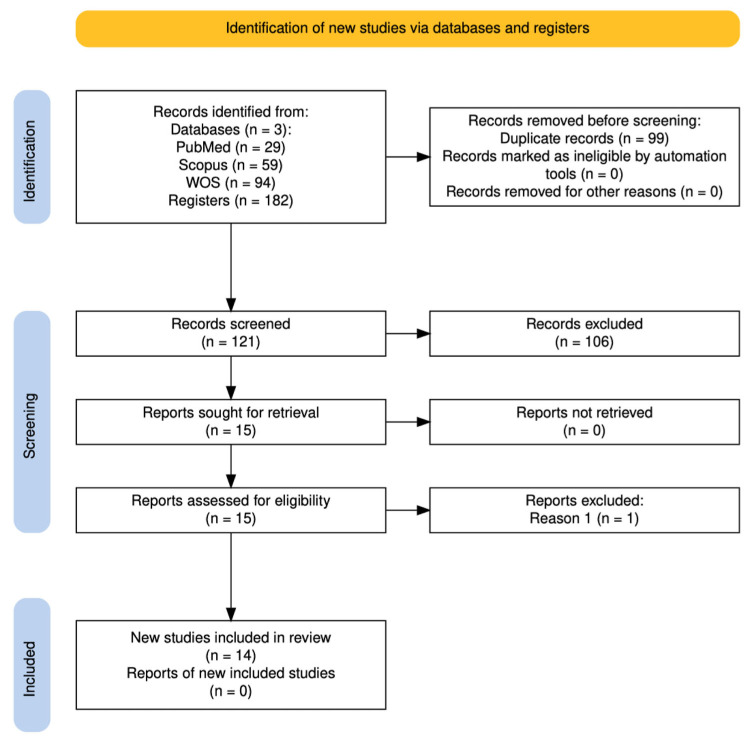
Flow chart of the study according to PRISMA criteria.

The database search identified 182 records (PubMed, 29; Scopus, 59; and Web of Science, 94). After removing 99 duplicates, 121 unique studies were screened by title and abstract, of which 106 were excluded for not meeting the predefined criteria. Fifteen full-text reports were then sought for retrieval, and all were successfully obtained. One study was excluded at this stage because it investigated extracellular vesicles derived from PRP rather than PRP itself [30]. Ultimately, 14 studies [31,32,33,34,35,36,37,38,39,40,41,42,43,44] fulfilled the eligibility criteria and were included in the qualitative synthesis (Figure 1).

### 3.2. Summary of Preclinical Evidence: Effects and Mechanisms of Platelet-Rich Plasma (PRP) in Models of Liver Disease

The methodological and outcome diversity of the 14 included preclinical studies [31,32,33,34,35,36,37,38,39,40,41,42,43,44] is detailed in Table S1. Building upon this structured overview, the following sections analyze the aggregated evidence across several thematic domains.

#### 3.2.1. Experimental Animals, Sex, and Sample Size

Most experiments used albino rat strains such as Wistar, Sprague–Dawley, or Swiss albino rats, while mice were employed specifically in a parasitic liver fibrosis model using *Schistosoma mansoni* [39]. Male animals were used preferentially across the majority of studies [31,32,33,34,35,38,40,41,42,43]. Several investigations did not explicitly report animal sex [37,39], and one study used female rats exclusively [36]. No study included mixed-sex experimental groups.

Sample sizes varied substantially between studies, ranging from small-scale experiments with 24–32 animals divided into multiple groups [31,36,40,43] to large, multi-arm designs including up to 162–180 animals [37,39]. Group sizes typically ranged from 6 to 10 animals per experimental group, with larger cohorts observed in studies evaluating combination therapies or multiple treatment timings [37,39]. This variability in sample size and group balance may have influenced statistical power and outcome robustness across studies.

#### 3.2.2. Experimental Models and Types of Liver Injury

The animal models represented a wide spectrum of liver injury mechanisms. Cholestatic fibrosis and cirrhosis induced by bile duct ligation (BDL) were employed in several rat studies to model portal hypertension [37,38]. Parasitic liver fibrosis was modeled using *Schistosoma mansoni* infection in mice 329]. Chemically induced hepatotoxicity and fibrosis were widely used and included carbon tetrachloride (CCl_4_), dimethylnitrosamine (DMN), thioacetamide (TAA), diclofenac sodium, cisplatin, and lead nitrate exposure [21,22,23,24,25,26,27,28,29,30,31,32,33,34,35,36,40,41,42,43]. Additional models incorporated whole-body gamma (γ)-radiation-induced hepatotoxicity [35,42] and liver regeneration following 70% partial hepatectomy [41,44].

This diversity allowed evaluation of PRP across fibrotic, toxic, inflammatory, oxidative, and regenerative contexts, although it also introduced considerable heterogeneity between studies.

#### 3.2.3. Effects of PRP and Related Hemocomponents on Hepatic Fibrosis and Extracellular Matrix Remodeling

Across most fibrosis-driven models, PRP or related hemocomponents administration resulted in a reduction in collagen deposition and fibrotic area, as demonstrated by histological staining (e.g., Masson’s trichrome, Sirius Red) and morphometric analyses [32,33,35,36,39,42,43]. Several studies reported decreased expression of profibrotic markers, including transforming TGF-β and α-SMA, suggesting attenuation of hepatic stellate cell activation [32,34,38,39].

However, antifibrotic effects were not uniform. In one stereological study, PRP supernatant reduced connective tissue volume but was associated with a significant reduction in hepatocyte number and volume and failed to improve biochemical liver function, resulting in an overall negative functional interpretation [31]. In parasitic fibrosis models, PRP effects were strongly dependent on administration route and timing, with intrahepatic delivery at later disease stages showing neutral or unfavorable outcomes [39].

#### 3.2.4. Hepatocellular Injury and Biochemical Liver Function

PRP (or PRP supernatant) treatment was generally associated with improvements in biochemical markers of liver injury. Decreases in serum alanine aminotransferase (ALT), aspartate aminotransferase (AST), alkaline phosphatase (ALP), and gamma-glutamyl transferase (GGT) were consistently reported in toxin-, drug-, and radiation-induced hepatotoxicity models [32,33,34,35,40,42,43]. Improvements in albumin levels and total protein synthesis were also documented in several studies [33,42].

Nonetheless, discrepancies between histological and biochemical outcomes were observed. In certain models, PRP-induced structural improvements were not accompanied by corresponding normalization of liver enzymes, highlighting context-dependent functional effects [31,41].

#### 3.2.5. Oxidative Stress, Inflammation, and Apoptotic Signaling

A consistent finding across multiple studies was the antioxidant and anti-inflammatory effect of PRP. Platelet-rich plasma administration significantly reduced oxidative and nitrosative stress markers, including malondialdehyde (MDA), nitric oxide, and peroxynitrite, while restoring endogenous antioxidant defenses such as glutathione (GSH), superoxide dismutase (SOD), catalase (CAT), glutathione S-transferase (GST), and NAD(P)H quinone oxidoreductase 1 (NQO1) activity [33,34,35,40,42,43].

Inflammatory signaling pathways, including NF-κB activation, macrophage infiltration, interleukin-8 (IL-8) expression, and macrophage inflammatory protein-1α (MIP-1α) concentrations, were attenuated following PRP treatment [32,33,38]. Reduced apoptotic activity was evidenced by downregulation of caspase-3 and upregulation of anti-apoptotic markers such as B-cell lymphoma 2 (Bcl-2) [32,34].

#### 3.2.6. Liver Regeneration and Proliferative Responses

In regeneration-focused models, PRP enhanced hepatocyte proliferation and liver mass recovery. Increased expression of proliferation markers, including Ki-67 and proliferating cell nuclear antigen (PCNA), as well as improved liver-to-body weight ratios, were reported following PRP administration after partial hepatectomy [36,44]. Activation of key regenerative pathways, including protein kinase B (Akt), extracellular signal-regulated kinases 1/2 (ERK1/2), and signal transducer and activator of transcription 3 (STAT3), was also demonstrated [42,44].

However, not all regenerative models showed histological improvement. In one study, PRP reduced oxidative stress but failed to improve histopathological regeneration indices (e.g., ductal proliferation), suggesting a dissociation between cytoprotective and regenerative effects [41].

#### 3.2.7. Angiogenic, Lymphangiogenic, and Mechanistic Effects

Mechanistic analyses were limited to a subset of studies. PRP-induced enhancement of lymphangiogenesis through vascular endothelial growth factor-C/vascular endothelial growth factor receptor-3 (VEGF-C/VEGFR-3) signaling was demonstrated in a BDL-induced portal hypertension model, resulting in improved lymphatic drainage and reduced portal pressure; these effects were abolished by VEGFR-3 inhibition (MAZ-51) [38]. PRP-associated angiogenic effects mediated by VEGF upregulation were also observed in drug-induced hepatotoxicity models [34].

Additional mechanisms for PRP and related hemocomponents included activation of ERK1/2 and Akt signaling pathways [42,44], regulation of microRNA-21 (miR-21) expression [35], and modulation of immune-related transcriptomic pathways [38].

#### 3.2.8. PRP as Monotherapy Versus Combination Therapy

PRP or related hemocomponents used as monotherapy produced heterogeneous outcomes, ranging from positive to neutral or context-dependent effects [31,37,39,41]. In contrast, combination therapies consistently demonstrated superior efficacy. PRP combined with mesenchymal stem cells (MSCs) and/or recombinant human hepatocyte growth factor (rh-HGF) produced marked synergistic antifibrotic and functional improvements in cholestatic cirrhosis models [37]. Similarly, PRP combined with praziquantel (PZQ) enhanced antifibrotic outcomes in parasitic liver fibrosis [39], and PRP combined with low-molecular-weight chitosan showed greater protection against radiation-induced hepatotoxicity than PRP alone [35].

#### 3.2.9. Follow-Up

Across the included studies, follow-up duration showed substantial heterogeneity, ranging from ultra-short mechanistic assessments to prolonged experimental observation periods. Follow-ups extended from minutes to 24 h in acute regeneration models, such as the study by Matsuo et al. [44], to long-term designs of up to 14 weeks in chronic parasitic fibrosis models [39]. This variability reflects differences in experimental objectives, liver injury models, and the intended role of PRP or related hemocomponents, whether as an acute hepatoprotective agent, an antifibrotic intervention, or a regenerative stimulus.

Very short follow-ups, limited to hours or one week, were primarily used in hepatectomy models to capture early regenerative signaling and platelet-mediated molecular events [41,44]. While suitable for mechanistic insights, these designs were insufficient to evaluate sustained tissue regeneration, fibrosis modulation, or long-term functional recovery. Short to intermediate follow-ups of 3–5 weeks were commonly applied in acute and subacute hepatotoxicity models, allowing demonstration of PRP-mediated improvements in liver enzymes, oxidative stress, inflammatory markers, and early histological changes [32,34,35,40,44]. However, these timeframes remained limited for assessing the durability of antifibrotic effects.

Intermediate to long follow-ups of 6–8 weeks were mainly used in cholestatic and chronic toxic fibrosis models, providing a more balanced temporal window to evaluate both biochemical and histological outcomes after established injury [33,37,43]. The longest follow-ups, extending from 10 to 14 weeks, were employed in studies of chronic chemical or parasitic fibrosis and were particularly informative in revealing time-dependent and context-dependent effects of PRP, including cases where histological improvement did not translate into functional recovery or where late administration produced neutral or adverse outcomes [31,36,39].

Overall, follow-up duration was closely aligned with study aims but remained a consistent limitation across the literature. No study evaluated long-term outcomes after PRP withdrawal or assessed relapse, fibrosis progression, or sustained functional benefit, which restricts conclusions regarding the durability and translational relevance of PRP effects in liver disease models.

#### 3.2.10. Overall Interpretation of PRP Impact

Overall, PRP or related hemocomponents demonstrated multifaceted hepatoprotective properties, including antifibrotic, antioxidant, anti-inflammatory, anti-apoptotic, and pro-regenerative effects across multiple experimental liver injury models [32,33,34,35,38,42,43,44]. However, these effects were highly context-dependent and influenced by animal model, disease stage, administration route, and concomitant therapies.

Taken together, the findings suggest that PRP is more consistently effective as an adjunctive therapy rather than as a uniform standalone intervention, underscoring the need for standardized PRP characterization and more rigorous mechanistic investigations to improve translational relevance.

The mechanistic and context-dependent effects of PRP, highlighting model-specific responses and methodological heterogeneity across experimental liver disease models, are summarized in Table 2. Furthermore, a heatmap summarizing the direction and consistency of PRP effects across biological domains and liver disease models is shown in Table 3. Interestingly, this heatmap depicts the direction and consistency of PRP effects within individual biological domains. These domain-specific effects do not necessarily translate into an overall beneficial outcome, which varied across studies and is summarized separately in Table S1.

### 3.3. Critical Evaluation of PRP Preparation Protocols

Beyond biological outcomes, a critical limitation of the current evidence lies in the inadequate methodological reporting of PRP preparation and characterization. An analysis of the reviewed studies reveals widespread and significant deficiencies in methodological reporting across all critical domains for PRP preparation and characterization. In the blood collection and pre-processing phase, while the anticoagulant type was commonly specified, essential parameters such as the exact time to centrifugation and the centrifugation temperature were almost universally not reported [33,34].

The total blood volume and the blood-to-anticoagulant ratio were also inconsistently provided. For PRP preparation, all studies employed a manual double-centrifugation method, with the exception of Matsuo et al. [44], which employed washed platelet preparations, but the reporting of centrifugation parameters was critically insufficient. Most failed to report the relative centrifugal force (× *g*), providing only revolutions per minute (RPM) without the necessary rotor radius for conversion, rendering the protocols non-reproducible [34,35,37,40,42,43]. The final PRP volume was another frequently omitted detail.

Cellular characterization was severely underreported. Although some studies provided a platelet concentration in the final PRP product, very few reported complete basal cell counts for both whole blood and PRP, and none included measures of variability like standard deviation [31,33,38,43]. Calculations for platelet enrichment factors and yield percentages were absent, and the analytical device used for cell counting was often unspecified. For biochemical characterization and protein enrichment controls, the reporting was nearly non-existent. None of the studies quantified soluble mediators such as growth factors or cytokines, and the use of negative or positive controls for protein release was not mentioned [31,32,33,34,35,36,37,38,39,40,41,42,43,44].

The description of platelet activation for the assay or for therapeutic application was either missing or critically vague. Several studies did not clarify if the PRP was activated prior to application [33,36,39,40], and among those that did, details on the activating agent, concentration, and incubation time were sparse [34,35,37,38]. A recurring major limitation was the use of only the PRP supernatant or filtered releasate instead of the complete platelet concentrate, which deviates from the definition of true PRP and confounds the interpretation of therapeutic effects [31,34,35,42,43].

Regarding experimental application, although the treated condition, administered volume, and delivery route were generally reported, the activation status of PRP was frequently unclear, and the rationale for non-activation was never explicitly stated. Moreover, no study described systematic monitoring for adverse effects following administration. Across the preclinical literature, routes of administration of platelet-rich plasma (PRP) and related hemocomponents were highly heterogeneous and model dependent. Intraperitoneal (IP) injection was the most commonly used approach in toxic, fibrotic, and post-hepatectomy models, typically involving repeated dosing schedules over several weeks [31,33,36,37,41]. Subcutaneous (SC) administration was also frequently employed, particularly in chemically or radiation-induced hepatotoxicity models, often using PRP supernatants or releasates rather than intact PRP [32,34,35,42,43]. Less commonly, intrahepatic (IH) injection was applied as a local delivery strategy, generally as a single dose, with variable or context-dependent outcomes [39]. In surgical regeneration settings, more direct vascular routes were explored, including portal vein infusion of washed platelets after partial hepatectomy and intramesenteric vein injection of platelet-derived supernatants [31,44]. Overall, the marked variability in administration routes, dosing regimens, activation practices, and product types, together with limited safety and methodological reporting, precludes standardized interpretation and cross-study comparison [31,32,33,34,35,36,37,38,39,40,41,42,43,44].

This collective lack of standardized reporting on mandatory items—particularly centrifugation parameters, complete cellular counts, and activation protocols—results in very low methodological quality and severely limits the reproducibility, comparability, and mechanistic interpretation of findings across these preclinical studies [31,32,33,34,35,36,37,38,39,40,41,42,43,44] (Table S2). The overall merit of the evidence generated is consequently diminished, highlighting an urgent need for adherence to comprehensive reporting guidelines in future PRP research.

**Figure 2:**
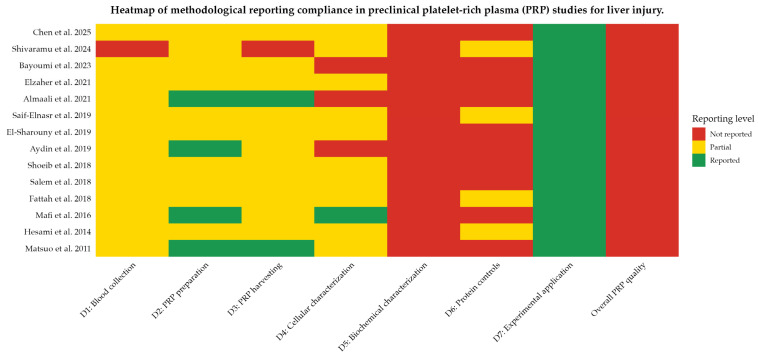
Heatmap of methodological reporting compliance in preclinical platelet-rich plasma (PRP) studies for liver injury. Rows represent individual studies ordered chronologically from most recent to oldest, and columns correspond to methodological domains D1–D7 and overall PRP quality. Color coding indicates the level of reporting (red, not reported; yellow, partially reported; green, reported). Overall PRP quality was classified as very low for all studies, as none fulfilled the mandatory reporting criteria across domains D1–D6. This uniform classification supports the narrative synthesis and explains the infeasibility of quantitative meta-analysis [31,32,33,34,35,36,37,38,39,40,41,42,43,44].

The methodological heterogeneity and overall low quality of reporting across the included studies are summarized in Figure 2. As illustrated by the heatmap, deficiencies were pervasive across all mandatory domains related to PRP preparation and characterization (D1–D6), with most studies reporting only isolated methodological elements and failing to provide the minimum information required for reproducibility.

Although the experimental application domain (D7) was generally described, critical pre-analytical, analytical, and biochemical variables were inconsistently or not reported, resulting in a uniform classification of very low overall PRP quality. This systematic lack of standardized reporting underscores the limited comparability of existing preclinical evidence and precludes meaningful quantitative synthesis.

Across the included studies, the platelet-based interventions were heterogeneous and could be categorized into three main product types: intact platelet-rich plasma (PRP) with viable platelets at the time of administration [33,36,38,39,40,44], platelet-derived supernatants or releasates obtained after activation and removal of platelet membranes [31,32,34,35,42,43], and platelet lysates generated through freeze–thaw procedures [37].

Notably, only a subset of studies administered intact PRP, whereas a comparable number relied exclusively on platelet-derived supernatants or lysates, limiting direct comparability across experimental models. Furthermore, it was not possible to classify PRP products according to the simplified Dohan-Ehrenfest classification of platelet concentrates [45,46], as platelet concentrations were not consistently reported across all studies and none provided quantitative data on leukocyte content [31,32,33,34,35,36,37,38,39,40,41,42,43,44]. The lack of standardized reporting of key compositional parameters precluded differentiation between leukocyte-rich PRP (L-PRP) and leukocyte-poor PRP (pure PRP (P-PRP)) formulations and represents a major source of methodological heterogeneity within the preclinical literature.

### 3.4. Assessment of Risk of Bias for the Included Studies

**Figure 3:**
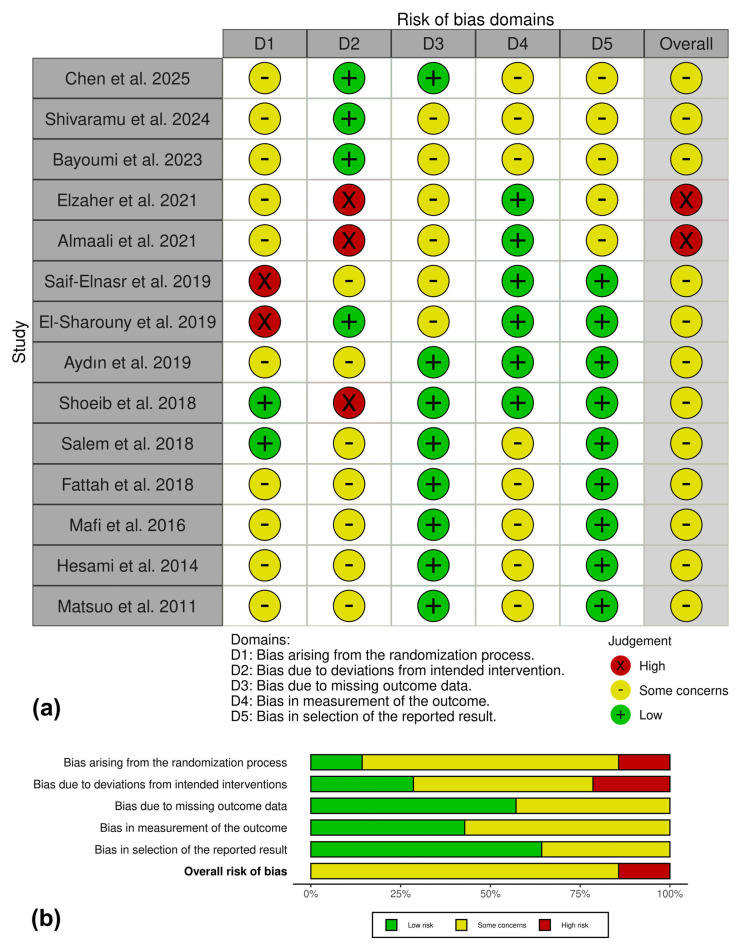
Risk of bias assessment of the included studies using the ROB 2.0 tool. (**a**) Traffic light plot summarizing the risk of bias judgments across the five ROB 2.0 domains for each included study. Each domain was judged as low risk (green), some concerns (yellow), or high risk (red), according to the ROB 2.0 criteria adapted for preclinical experimental studies [31,32,33,34,35,36,37,38,39,40,41,42,43,44]. (**b**) Summary plot showing the proportion of studies rated as low risk, some concerns, or high risk for each ROB 2.0 domain and for the overall risk of bias.

Using the ROB 2.0 tool, the overall risk of bias across the 14 included studies was predominantly judged as some concerns, with a smaller proportion of studies classified as high risk and no study consistently rated as low risk across all domains (Figure 3a). Concerns were most frequently related to the randomization process (Domain 1), reflecting limited reporting of sequence generation and allocation procedures, which is common in preclinical experimental studies. In contrast, bias due to missing outcome data (Domain 3) and bias in selection of the reported result (Domain 5) were more frequently judged as low risk across studies.

A domain-level summary demonstrated that deviations from intended interventions (Domain 2) accounted for most of the high-risk judgments, whereas bias in outcome measurement (Domain 4) was generally rated as low risk or associated with some concerns, mainly due to the absence of explicit assessor blinding in histological and immunohistochemical analyses (Figure 3b). Overall, while methodological limitations were identified, particularly in reporting transparency, the ROB 2.0 assessment did not reveal widespread high risk of bias across the body of evidence.

This systematic review provides a comprehensive synthesis of the preclinical evidence evaluating platelet-rich plasma and related platelet-derived hemocomponents in experimental models of liver injury and regeneration. Overall, the available data indicate that platelet-based interventions can exert hepatoprotective, antifibrotic, and regenerative effects across a wide range of experimental contexts, including toxic, cholestatic, parasitic, radiation-induced, and surgical models [31,32,33,34,35,36,37,38,39,40,41,42,43,44]. However, these effects were highly context-dependent and varied according to the etiology and stage of liver injury, the route and timing of administration, and, critically, the specific platelet product used. Importantly, although many studies referred to their intervention as PRP, a substantial proportion administered platelet-derived supernatants, releasates, lysates, or washed platelet preparations rather than intact PRP containing viable platelets [31,32,34,35,37,42,43,44].

This heterogeneity in platelet-based products complicates direct comparison across studies and limits mechanistic interpretation, as these formulations differ fundamentally in cellular composition, bioactive mediator release kinetics, and biological behavior. Consequently, while beneficial effects were frequently reported, particularly, in combination therapies or specific experimental settings [37,39,42], the collective evidence underscores the need for cautious interpretation of translational conclusions derived from preclinical platelet-based interventions.

Importantly, interpretation of the present findings requires careful distinction between platelet-rich plasma as a living biological product and platelet-derived fractions that represent only partial systems of the original PRP. True PRP is a dynamic, cell-based hemocomponent containing viable platelets and, depending on the formulation, functional leukocytes, which together act as a biological model capable of sustained mediator release, cell–cell interactions, and time-dependent biological responses following activation and fibrin polymerization [47]. In contrast, platelet-derived supernatants and platelet lysates are acellular or cell-devoid products enriched in soluble mediators but lacking functional platelets and leukocytes, and therefore cannot reproduce the complex spatiotemporal signaling, cellular viability, and scaffold-mediated effects intrinsic to intact PRP [48,49]. Experimental evidence demonstrates that platelet activation, freeze–thaw procedures, or prolonged storage fundamentally alter mediator release kinetics, denaturation profiles, and biological behavior when compared with fresh PRP or platelet-rich gels [48,49].

Nevertheless, a generalized conceptual imprecision persists in the literature, whereby diverse platelet-derived hemocomponents are frequently labeled as “PRP,” likely reflecting inconsistent methodological reporting and insufficiently rigorous peer-review standards. This terminological ambiguity has contributed to substantial heterogeneity across preclinical studies and may partially explain divergent mechanistic interpretations attributed to PRP-based therapies.

Although the overall analysis of the included studies suggests a generally favorable biological response to PRP and related hemocomponents, this observation must be interpreted with caution [31,32,33,34,35,36,37,38,39,40,41,42,43,44]. The apparent efficacy reported across experimental models is confounded by the wide spectrum of platelet-based products evaluated and by their limited or absent characterization in most studies. Indeed, across the 14 included investigations, it is frequently not possible to determine with precision which biological entity was administered, as key compositional parameters, such as platelet concentration, leukocyte content, mediator concentration, activation status, and cellular viability, among others, were inconsistently reported or entirely omitted [31,32,33,34,35,36,37,38,39,40,41,42,43,44]. Notably, beneficial effects were observed even in studies employing xenogeneic platelet products [39], underscoring that the reported responses may, at least in part, reflect non-specific mediator-driven effects rather than true platelet-mediated biological activity.

Evidence from other fields, such as osteoarthritis [50] and bone regeneration [51], indicates that PRP does not consistently outperform placebo, particularly in well-controlled clinical trials, highlighting the context-dependent nature of platelet-based therapies. Although fewer negative or null studies exist in liver models, similar limitations are apparent. Several studies included in this review reported neutral or unfavorable outcomes, particularly when PRP was used as monotherapy, administered at late disease stages, or delivered intrahepatically in advanced fibrosis [31,36,39,41]. Moreover, the liver’s structural and functional complexity—including metabolic zonation, dual blood supply, and tightly regulated inflammatory and fibrotic responses—may pose unique challenges for PRP-based interventions, potentially limiting the uniform translation of regenerative effects observed in other tissues [31,38,39,44].

Recruitment of leukocytes, particularly circulating monocytes and their differentiation into hepatic macrophages, is a fundamental component of liver repair following toxic injury or surgical resection [52]. Within the studies included in this systematic review, direct quantitative assessment of monocyte trafficking was limited or absent. Nevertheless, several investigations provided indirect evidence on this reparative axis by evaluating inflammatory signaling pathways and macrophage-related readouts. Specifically, PRP and related hemocomponents were associated with attenuation of inflammatory signaling, including reduced nuclear factor kappa B (NF-κB) activation and decreased expression of pro-inflammatory mediators, as well as reduced macrophage infiltration, commonly assessed by CD68-positive cell density [32,33,38,43].

In parallel, modulation of inflammatory mediators such as interleukin-8 (IL-8) and macrophage inflammatory protein-1α (MIP-1α) was reported in selected models, further supporting an immunomodulatory rather than purely pro-inflammatory effect of platelet-based interventions [33,38]. Importantly, most studies failed to report leukocyte content within the administered product, precluding classification into leukocyte-rich versus leukocyte-poor PRP formulations and limiting mechanistic inference regarding the specific contribution of leukocytes or monocytes to the observed hepatic responses [31,32,33,34,35,36,37,38,39,40,41,42,43,44]. This lack of standardized cellular characterization represents a key limitation for interpreting immune-mediated mechanisms of PRP in liver regeneration.

From the authors’ perspective, these findings do not negate the therapeutic potential of platelet-based interventions but instead emphasize the need to revisit preclinical research in this field with more rigorous product characterization, improved reporting standards, and experimental designs that better approximate clinical reality. Future studies should prioritize standardized description of PRP and related hemocomponents and consider the use of larger animal models, such as rabbits [53,54,55], or naturally occurring clinical conditions, including chronic active inflammatory hepatitis in dogs [56,57,58,59,60], to enhance biological relevance and translational applicability.

In many pathological contexts, PRP and related platelet-derived hemocomponents should not be interpreted as stand-alone etiological therapies but rather as biological modulators that may complement standard or causal interventions. For instance, in parasitic liver disease models, such as schistosomiasis, parasite eradication necessarily depends on antiparasitic treatment (i.e., praziquantel), and the beneficial effects observed following co-administration of platelet-based products are more appropriately attributed to modulation of inflammation, granuloma remodeling, and tissue repair rather than to direct disease resolution [39].

Similarly, in studies evaluating mesenchymal stromal cells or growth factor–based strategies, platelet-derived products have frequently been used as delivery matrices or potentiating substrates, often with the explicit aim of enhancing cell survival, engraftment, or paracrine signaling, making it difficult to attribute therapeutic superiority to a single intervention [37,38]. These experimental designs, while biologically plausible, underscore the need for controlled comparative approaches to disentangle additive from synergistic effects.

From this perspective, future preclinical and translational studies should incorporate multi-arm or factorial designs directly comparing platelet-based products administered alone versus in combination with established therapies, while also accounting for product origin (autologous versus allogeneic) and ensuring rigorous characterization of the administered hemocomponent [31,32,33,34,35,36,37,38,39,40,41,42,43,44]. Such approaches would allow a more precise determination of whether intact PRP can function as an effective monotherapy in selected contexts or whether its primary translational value lies in its role as an adjuvant biological system.

The primary limitation of this systematic review arises from the characteristics of the available preclinical evidence rather than from the review process itself. Across the included studies, there was substantial heterogeneity in the platelet-based products evaluated, encompassing intact PRP, platelet-derived supernatants, lysates, and modified platelet preparations. Crucially, key compositional parameters—such as platelet concentration, leukocyte content, activation status, and cellular viability—were inconsistently reported or entirely absent in most studies.

This limitation is not unique to the present review, as numerous systematic reviews in both human and veterinary medicine have consistently identified inadequate reporting and poor characterization of PRP preparations as one of the main constraints of the published literature [61,62,63,64,65]. As a result, it was frequently not possible to determine with precision which biological entity was administered, precluding application of established PRP classification systems and limiting mechanistic interpretation. This lack of standardized product characterization represents a fundamental barrier to cross-study comparability and reproducibility.

Several expert-driven initiatives have proposed standardized nomenclature and coding systems for PRP to improve reproducibility and cross-study comparability, particularly in musculoskeletal disorders. For example, an expert consensus proposed a structured PRP classification and coding system based on platelet concentration, leukocyte content, and activation status [66]. However, such classification frameworks could not be applied to the present body of preclinical liver studies due to the pervasive lack of reporting of mandatory compositional parameters, reinforcing the need for improved methodological transparency in future hepatic PRP research.

A second limitation relates to methodological heterogeneity and translational scope. The included studies employed diverse animal models, injury etiologies, administration routes, dosing regimens, follow-up durations, and outcome measures, which collectively precluded quantitative meta-analysis and restricted inference to qualitative synthesis [67]. In addition, risk-of-bias assessments revealed predominantly “some concerns” or “high risk” judgments, driven mainly by insufficient reporting of randomization procedures, blinding, and prespecified analysis plans. Similar concerns regarding methodological transparency and reproducibility have been highlighted in previous systematic reviews of platelet-rich plasma applications across clinical and preclinical settings [61,62,63,64,65]. Finally, the exclusive reliance on small-animal experimental models limits direct extrapolation to clinical practice, as few studies addressed spontaneous or naturally occurring liver diseases. Future research would benefit from standardized reporting frameworks, rigorously controlled comparative designs, and the inclusion of larger animal models or clinically relevant veterinary conditions to enhance biological relevance and translational validity.

The available preclinical evidence suggests that PRP and related platelet-derived hemocomponents can exert biologically relevant effects in experimental models of liver injury [31,32,33,34,35,36,37,38,39,40,41,42,43,44]; however, the interpretation and translational value of these findings are constrained by substantial heterogeneity in product composition, limited methodological transparency, and variable experimental contexts. A central conclusion of this review is that platelet-based interventions cannot be considered as uniform entities, as intact PRP represents a living biological system distinct from platelet-derived supernatants or lysates, each with different mechanistic implications.

To date, no controlled clinical trials evaluating PRP for liver injury or regeneration have been published, either in human or veterinary medicine. Consequently, no evidence-based recommendations exist regarding optimal PRP volume, platelet dose, or dose scaling relative to liver size or lesion area. The preclinical literature reviewed here shows substantial variability in administered volumes and routes, without standardized justification based on liver surface area, tissue mass, or platelet numbers. This lack of dose–response data represents a major translational gap that should be addressed in future experimental and early-phase clinical studies.

## 4. Conclusions

While favorable outcomes have been reported across diverse models, including in combination with established therapies, the lack of standardized characterization and reporting precludes definitive conclusions regarding efficacy, optimal formulation, or clinical applicability. Future research should prioritize rigorous description of platelet-based products, controlled comparative designs, and biologically relevant experimental models to clarify the role of PRP and related hemocomponents in hepatic regeneration and to support their rational translation into clinical and veterinary practice.

## Figures and Tables

**Table 1:** Minimum reporting checklist for PRP studies *.

Domain	Reporting Item	Specific Requirements	Mandatory/Recommended
1. Blood Collection and Pre-processing	Anticoagulant type and concentration	Specify anticoagulant used (e.g., ACD-A, sodium citrate) and its concentration.	Mandatory
	Total blood volume	Report volume of whole blood collected per animal (mL).	Mandatory
	Blood-to-anticoagulant ratio	Provide the exact ratio (e.g., 9:1).	Mandatory
	Time to centrifugation	Report elapsed time between venipuncture and start of centrifugation.	Mandatory
	Centrifugation temperature	Report centrifugation temperature (°C).	Mandatory
2. PRP Preparation	Method used	State whether a manual method or commercial kit was used; include product name and manufacturer.	Mandatory
	Centrifugation parameters	Report relative centrifugal force (× *g*), spin duration (min), number of spins, and temperature.	Mandatory
	Revolutions per minute (RPM) reporting	Do not report rpm alone. If rpm is used, provide rotor radius (cm) for × *g* conversion.	Mandatory
3. PRP Harvesting	Harvesting technique	Describe technique used (e.g., buffy coat aspiration, plasma supernatant extraction).	Mandatory
	Final PRP volume	Report final volume of PRP obtained (mL).	Mandatory
4. Cellular Characterization	Cell counts	Provide platelet, WBC, and RBC counts for both whole blood and PRP, ideally with variability (e.g., SD).	Mandatory
	Enrichment and yield	Report platelet enrichment factor (PRP ÷ whole blood) and % platelet yield.	Mandatory
	Analytical device	Specify hematology analyzer used; state whether same device was used for both measurements.	Mandatory
5. Biochemical Characterization	Soluble mediator quantification	Quantify growth factors or cytokines (e.g., TGF-β1, PDGF-BB, IL-1β) when possible.	Recommended
	Activation or lysis for assay	Describe physiological activation (e.g., CaCl_2_, thrombin) or lysis (e.g., detergent, freeze–thaw) used for protein release.	Recommended
6. Protein Enrichment Controls	Negative control	Measure mediator levels in platelet-poor plasma and/or native plasma.	Recommended
	Positive control	Include PRP lysed with detergent or ≥3 freeze–thaw cycles to determine maximal protein release.	Recommended
	Activation clarification	Clarify that lysis is not platelet activation but cellular destruction (including leukocytes).	Recommended
7. Clinical or experimental Application (in vivo)	Treated tissue or condition	State anatomical site or clinical indication (e.g., tendon lesion, osteoarthritis).	Mandatory
	Dose and number of applications	Report volume administered per dose (mL) and total number of doses.	Mandatory
	PRP activation status	Indicate whether PRP was activated before application.	Mandatory
	Activation details	If activated, report activating agent, concentration, and incubation time.	Mandatory
	Non-activation rationale	If not activated, state explicitly and provide the rationale.	Mandatory
	Delivery method	Specify route of administration and technique (e.g., US-guided injection, scaffold delivery).	Mandatory
	Monitoring and adverse effects	Report duration of follow-up and all observed adverse events, local or systemic.	Mandatory

* Table adapted from reference [26].

**Table 2:** Mechanistic and context-dependent effects of platelet-rich plasma and related hemocomponents in experimental liver disease models.

Biologic Domain	Evidence-Supported Effects (Model-Dependent)	Liver Disease Models (Key Examples)	Key References
Growth Factors & Signaling	Release of Vascular Endothelial Growth Factor (VEGF), Hepatocyte Growth Factor (HGF), Insulin-like Growth Factor-1 (IGF-1), Platelet-Derived Growth Factor (PDGF); activation of Extracellular signal–Regulated Kinase (ERK), Protein Kinase B (Akt), and Phosphoinositide 3-Kinase/Akt (PI3K/Akt) pathways (reported in selected models).	Partial hepatectomy, Toxic hepatotoxicity (i.e., lead nitrate/γ-radiation)	[32,42,44]
Vascular Endothelial Growth Factor-C/Vascular Endothelial Growth Factor Receptor-3 (VEGF-C/VEGFR-3) Axis	Platelet-derived VEGF-C promotes lymphangiogenesis, improves lymphatic drainage, and reduces portal hypertension.	Cholestatic cirrhosis & Portal Hypertension (Bile Duct Ligation—BDL)	[38]
Immunomodulation & Inflammation	Reduction of Interleukin-8 (IL-8), Nuclear Factor kappa B (NF-κB), and CD68-positive macrophage infiltration; suppression of pathological intrahepatic angiogenesis (context-dependent).	Toxic fibrosis (Dimethylnitrosamine—DMN, Thioacetamide—TAA), Cholestatic cirrhosis (BDL)	[32,33,38]
Antioxidant & Anti-apoptotic Effects	Reduced lipid peroxidation (Malondialdehyde/Thiobarbituric Acid Reactive Substances/Nitric Oxide—MDA/TBARS/NO); restoration of Glutathione (GSH), Superoxide Dismutase (SOD), Catalase (CAT), and NAD(P)H Quinone Dehydrogenase 1 (NQO1); upregulation of B-cell Lymphoma 2 (Bcl-2).	Toxic & Drug-induced hepatotoxicity (Cisplatin, Diclofenac, CCl_4_, Lead nitrate/γ-radiation, TAA)	[32,33,35,40,42,43]
Fibrosis Modulation	Reduced collagen deposition, Alpha-Smooth Muscle Actin (α-SMA), and Transforming Growth Factor-beta (TGF-β) expression; possible dissociation between histological and functional recovery.	Toxic fibrosis (CCl_4_, DMN), Parasitic fibrosis (*Schistosoma mansoni*)	[31,32,36,39,43]
Toxic/Radiation Injury Models	Hepatoprotective effects against specific toxins and radiation.	Drug-induced (Cisplatin, Diclofenac), Chemical-induced (Lead nitrate), Radiation-induced (γ-radiation)	[34,35,40,42]
Liver Regeneration	Early enhancement of hepatocyte proliferation and regenerative signaling after hepatectomy; effects limited to the acute/subacute phase.	Surgical regeneration (70% Partial hepatectomy)	[41,44]
Combination Therapy	Synergistic effects with other biologics or drugs; PRP is more effective as an adjuvant than as monotherapy.	Cholestatic cirrhosis (BDL + ADMSCs/rh-HGF), Parasitic fibrosis (*S. mansoni* + Praziquantel)	[37,39]
Key Limitations	High heterogeneity in PRP preparation, dosing, timing, administration routes, and follow-up; effects are model- and time-dependent.	Across all included models	[31,32,33,34,35,36,37,38,39,40,41,42,43,44]

**Table 3:** Heatmap of reported PRP effects across experimental liver injury models.

Biological Domain	Model					
	Partial Hepatectomy	Acute Toxic Injury (i.e., Cisplatin, Drug)	Radiation Injury	Chronic Toxic Fibrosis (i.e., CCl_4_, DMN, TAA)	Cholestatic Fibrosis & PH (BDL)	**Parasitic Fibrosis (*S. mansoni*)**
Oxidative Stress	🟨	🟩	🟩	🟨	🟨	🟨
Inflammation	⬜	🟩	🟩	🟨	🟨	🟨
Apoptosis	⬜	🟩	🟩	🟨	⬜	⬜
Fibrosis (ECM)	⬜	⬜	⬜	🟨	🟨	🟨 (IP)/🟥 (IH)
Hepatocyte Proliferation	🟩	⬜	⬜	🟥	⬜	⬜
Biochemical Recovery	🟨	🟩	🟩	🟨	🟨	🟨
Angiogenesis	⬜	🟨	⬜	🟨	🟨	⬜
Lymphangiogenesis	⬜	⬜	⬜	⬜	🟩	⬜
Portal Pressure	⬜	⬜	⬜	⬜	🟩	⬜
Efficacy of Combination Therapy	⬜	⬜	🟩 *	🟨 *	🟩	🟩

Colors indicate the direction and consistency of reported outcomes within the reviewed studies, not the magnitude of the effect. 🟩 Consistent positive effect reported; 🟨 Variable, context- or time-dependent effect; 🟥 Null or unfavorable effect reported; ⬜ Outcome not evaluated in the model. IP = intraperitoneal route; IH = intrahepatic route (late administration). * Combination with chitosan [35], adipose-derived stem cells [37], or praziquantel [39].

## Data Availability

The original contributions presented in this study are included in the article/Supplementary Materials. Further inquiries can be directed to the corresponding author.

## Supplementary Materials

The following supporting information can be downloaded at: https://www.mdpi.com/article/10.3390/ijms27021013/s1.

## Abbreviations

The following abbreviations are used in this manuscript:

AAV	Adeno-Associated Virus
ADMSCs	Adipose-Derived Mesenchymal Stem Cells
ALP	Alkaline Phosphatase
ALT	Alanine Aminotransferase
AST	Aspartate Aminotransferase
Bcl-2	B-Cell Lymphoma 2
BDL	Bile Duct Ligation
CAT	Catalase
CCl_4_	Carbon Tetrachloride
CI	Confidence Interval
DMN	Dimethylnitrosamine
DX	Dexpanthenol
ELISA	Enzyme-Linked Immunosorbent Assay
ERK	Extracellular Signal-Regulated Kinase
GGT	Gamma-Glutamyl Transferase
GO	Gene Ontology
GSEA	Gene Set Enrichment Analysis
GSH	Reduced Glutathione
H&E	Hematoxylin and Eosin
HAI	Histological Activity Index
HGF	Hepatocyte Growth Factor
IH	Intrahepatic
IP	Intraperitoneal
KEGG	Kyoto Encyclopedia of Genes and Genomes
LDH	Lactate Dehydrogenase
LYVE-1	Lymphatic Vessel Endothelial Hyaluronan Receptor 1
MAZ-51	VEGFR-3 Tyrosine Kinase Inhibitor
MDA	Malondialdehyde
METAVIR	Meta-Analysis of Histological Data in Viral Hepatitis
MMP	Matrix Metalloproteinase
MSCs	Mesenchymal Stem Cells
NF-κB	Nuclear Factor Kappa B
NQO1	NAD(P)H Quinone Dehydrogenase 1
PCNA	Proliferating Cell Nuclear Antigen
PL	Platelet Lysate
PRG	Platelet-Rich Gel
PRP	Platelet-Rich Plasma
PZQ	Praziquantel
ROB 2.0	Revised Cochrane Risk of Bias Tool for Randomized Trials
ROS	Reactive Oxygen Species
SMA	Alpha-Smooth Muscle Actin
SOD	Superoxide Dismutase
STAT3	Signal Transducer and Activator of Transcription 3
TAA	Thioacetamide
TBARs	Thiobarbituric Acid Reactive Substances
TGF-β	Transforming Growth Factor Beta
TIMP	Tissue Inhibitor of Metalloproteinases
TQ	Thymoquinone
VEGF	Vascular Endothelial Growth Factor
VEGFR	Vascular Endothelial Growth Factor Receptor
