# Supplementary material for: Molecular and Regenerative Effects of Platelet-Rich Plasma and Related Hemocomponents in Animal Models of Liver Injury—A Systematic Review
Source: Int J Mol Sci. 2026 Jan 20;27(2):1013. doi: 10.3390/ijms27021013 (PMC12841623; doi:10.3390/ijms27021013)
Supplement: Supplementary file 1 [file ijms-27-01013-s001.zip › ijms-4081437-supplementary.pdf]

International Journal of *Molecular Sciences*

MDPI

Supplementary Materials

Table S1. Preclinical in vivo studies evaluating the effects of platelet-rich plasma (PRP) in experimental liver injury, fibrosis, and regeneration models.

Authors and year	Animal model and n	Aims	Design of the study and time follow-up	Outcome variables	Findings	Limitations	Overall PRP impact
Chen et al. 2025 [38]	Sprague–Dawley male rats, bile duct ligation (BDL). n = 50 (10/group)	To investigate the role of lymphangiogenesis and platelet-mediated lymphatic regulation in a preclinical model of portal hypertension	A controlled in vivo experimental study in rats employing a BDL model of portal hypertension with pharmacological and biological interventions targeting lymphangiogenesis. Portal pressure and lymphangiogenesis assessed across sham-operated control, BDL–vehicle, BDL + adeno-associated virus vector encoding vascular endothelial growth factor-C (AAV-VEGF-C), BDL + platelet-rich plasma (PRP), and BDL + PRP +	Portal pressure (hemodynamic measurement); lymphangiogenesis (number and area of LYVE-1–positive lymphatic vessels; VEGF-C and VEGFR-3 expression); liver fibrosis (Masson's trichrome and Sirius Red staining; transforming growth factor beta (TGF-β), alpha-smooth muscle actin (α-SMA), fibronectin expression); inflammation (CD68-positive macrophage infiltration); angiogenesis (CD31, VEGF-A, von Willebrand factor (vWF), matrix metalloproteinase 2 (MMP-2) expression); mesenteric vascular remodeling (superior mesenteric artery wall thickness); transcriptomic changes in lymphatic vessels (differentially expressed genes and	PRP-induced platelet activation enhanced lymphangiogenesis via VEGF-C/vascular endothelial growth factor receptor 3 (VEGFR-3), increased lymphatic drainage, and reduced portal pressure. Secondary effects included reduced liver fibrosis, inflammation, angiogenesis, and mesenteric vascular remodeling. Transcriptomic analysis of lymphatic vessels identified platelet-related and immune pathways as key mechanisms. VEGFR-3	The study had several limitations. The model is specific to acute cholestatic cirrhosis, which restricts the generalizability of the findings to other etiologies of portal hypertension. Furthermore, the sample size per group was relatively small and became unbalanced by the study's end, key methodological details such as randomization and blinding procedures were not fully detailed, and the evaluation did not include long-term outcomes or dose–response effects.	Positive (PRP-induced platelet activation enhanced lymphangiogenesis, and increased lymphatic drainage, and reduced portal pressure)

			MAZ-51 groups. Follow-up: 4 weeks	GO/KEGG/GSEA enrichment); platelet-related markers (CD41 expression)	inhibition (MAZ-51) abolished these effects		
Shivaramu et al. 2024 [37]	Rat, BDL. n = 180 (20/group). Sex of the animals not specified	To evaluate the hepatoprotective effects of adipose-derived mesenchymal stem cells (ADMSCs) combined with PRP and recombinant human hepatocyte growth factor (rh-HGF) in a BDL-induced cirrhosis model	Controlled in vivo study with 9 groups: Sham, BDL control, and BDL + various combinations of ADMSCs, PRP, and rh-HGF. Treatment from week 2 to 6 post-surgery. Follow-up: 6 weeks	Clinical signs, body weight, survival, biochemistry (aspartate aminotransferase (AST), alanine aminotransferase (ALT), alkaline phosphatase (ALP), gamma-glutamyl transferase (GGT), bilirubin, albumin, HGF, MMP-2, tissular inhibitor of matrix metalloproteinase 2 (TIMP-2), oxidative stress), histopathology (HAI, Knodell, METAVIR scores, fibrosis %), proliferating cell nuclear antigen (PCNA) proliferation, ADMSC tracking	PRP alone: No significant hepatoprotection. PRP + rh-HGF: Moderate improvement. ADMSCs + PRP: Significant synergy. ADMSCs + PRP + rh-HGF: Optimal synergistic effect—greatest fibrosis reduction (79.7%), improved biochemistry, histology, and antioxidant capacity	Large sample size, comprehensive multi-parameter assessment, clear demonstration of synergistic therapy. PRP alone ineffective, model limited to cholestatic BDL, no deep mechanistic insight into PRP's role in combination	Neutral (PRP alone). Positive (PRP in combination)
Bayoumi et al. 2023 [39]	Male CD-1 Swiss albino mice (n = 162, 6-12 mice/group) with *Schistosoma mansoni*–induced hepatic fibrosis experimental model.	To evaluate the hepatoprotective and therapeutic effects of PRP on liver fibrosis in *Schistosoma mansoni*–infected mice, to determine the optimal route and timing of PRP administration, and to assess the potential synergistic effect of combined PRP and praziquantel	A controlled in vivo experimental study in mice using a *Schistosoma mansoni*–induced liver fibrosis model, including 14 experimental groups: 6 non-infected control groups (sham, PRP intraperitoneal, PRP intrahepatic, PZQ, PRP intraperitoneal + PZQ, PRP intrahepatic + PZQ) and 8 infected groups (untreated infected control, PRP	Parasitological and histopathological outcomes, including hepatic granuloma number and diameter, fibrotic index (%) assessed by Masson's trichrome staining and image analysis; immunohistochemical expression of TGF-β1; comparative evaluation of early (12 weeks) and late (14 weeks) post-infection effects across treatment groups receiving platelet-rich plasma (intraperitoneal or intrahepatic), PZQ, or combined therapies.	PRP administration resulted in a reduction of hepatic granuloma number, granuloma diameter, fibrotic index, and TGF-β1 expression, with effects that were route- and time-dependent. The most pronounced antifibrotic effects were observed with intraperitoneal PRP, particularly when combined with PZQ and when administered at	PRP effects were heterogeneous and time-dependent, with inconsistent and occasionally adverse outcomes, particularly following intrahepatic administration at later stages. Mechanistic insight was limited mainly to TGF-β1 immunohistochemistry, without deeper molecular or pathway-level analyses. The model represents parasitic liver fibrosis only, limiting generalizability to toxic,	Positive (PRP + PZQ)<br>Neutral to negative (PRP alone, especially via intrahepatic delivery)

		(PZQ) treatment	intraperitoneal, PRP intrahepatic at week 6, PRP intrahepatic at week 10, PZQ, PRP intraperitoneal + PZQ, PRP intrahepatic + PZQ at week 6, and PRP intrahepatic + PZQ at week 10), with early (12 weeks) and late (14 weeks) outcome assessments. Follow-up: 14 weeks		earlier stages of infection. Intrahepatic PRP showed variable and sometimes unfavorable effects, including increased granuloma burden and fibrosis at later stages. Overall, PRP demonstrated context-dependent hepatoprotective effects, enhanced by combination therapy rather than monotherapy	metabolic, or cholestatic liver disease, and PRP monotherapy showed variable efficacy, supporting a predominantly adjunctive rather than standalone therapeutic role	
Elzaher et al. 2021 [36]	Adult female albino rats (n = 24, 6 rats/group) subjected to carbon tetrachloride (CCl_4)–induced liver fibrosis experimental model	To assess the histological and immunohistochemical effects of platelet-rich plasma on experimentally induced liver fibrosis in adult albino rats	A controlled in vivo experimental study in female albino rats using a CCl_4–induced liver fibrosis model, including four groups (control, CCl_4-induced fibrosis, spontaneous recovery after CCl_4 withdrawal, and CCl_4-induced fibrosis treated with PRP), with histological, immunohistochemical, morphometric, and statistical evaluation of liver tissue. Follow-up: 10 weeks	Histological assessment of liver architecture using hematoxylin and eosin staining; quantification of hepatic fibrosis by Masson's trichrome staining and morphometric analysis of collagen area percentage; immunohistochemical evaluation of hepatocellular proliferation using PCNA–positive cell count	CCl_4 administration induced marked hepatocellular injury and extensive hepatic fibrosis, while PRP treatment resulted in significant histological improvement, including restoration of hepatic architecture, a marked reduction in collagen deposition, and a significant increase in hepatocellular proliferation as evidenced by increased PCNA-positive cells; however, limited areas of fibrosis	The sample size was small, outcomes were limited mainly to histology without biochemical or functional liver parameters, and mechanistic insight was restricted to PCNA immunohistochemistry. Only female rats were used, and long-term outcomes or dose–response effects of PRP were not evaluated, limiting external validity and translational interpretation	Positive (PRP reduced collagen deposition and significantly increased hepatocellular proliferation)

					persisted despite PRP treatment		
Almaali et al. 2021 [40]	Male Wistar rats (n = 32) subjected to a diclofenac sodium–induced hepatotoxicity and nephrotoxicity experimental model. 10 rats/group	To investigate the protective effects of PRP against diclofenac sodium–induced hepatic and renal toxicity in rats by evaluating changes in biochemical markers of liver and kidney function	A controlled in vivo experimental study in male Wistar rats, including three groups (control, diclofenac sodium–treated, and diclofenac sodium + platelet-rich plasma), evaluating the protective effect of a single subcutaneous PRP dose against diclofenac-induced hepatotoxicity and nephrotoxicity over a 28-day treatment period, using biochemical assessment of liver and kidney function. Follow-up: 4 weeks	Biochemical markers of liver function, including serum aspartate aminotransferase (AST) and alanine aminotransferase (ALT); biochemical markers of kidney function, including blood urea nitrogen and serum creatinine levels, measured after 28 days of treatment to assess diclofenac-induced organ toxicity and the protective effect of platelet-rich plasma	Diclofenac sodium administration induced significant hepatotoxicity and nephrotoxicity, evidenced by marked increases in serum AST, ALT, and blood urea levels compared with controls. PRP co-administration significantly attenuated diclofenac-induced liver enzyme elevations and reduced blood urea levels, shifting biochemical parameters toward normal values, while serum creatinine levels were not significantly affected among groups, indicating a partial but consistent protective effect of PRP against drug-induced organ toxicity	Outcomes were limited to serum biochemistry without histological confirmation, mechanistic analyses, or assessment of oxidative stress markers. PRP dosing was single and not standardized by platelet concentration, long-term effects were not evaluated, and two animals were excluded from analysis due to PRP preparation, reducing effective sample size. The model reflects acute drug-induced toxicity, limiting extrapolation to chronic liver disease or fibrosis	Positive (PRP exerted a protective effect against diclofenac-induced hepatotoxicity)
Saif-Elnasr et al. 2019 [35]	Adult albino rats (n = 48 male, 6 rats/group), subjected to whole-body	To evaluate the therapeutic potential of platelet-rich plasma alone or in combination with	A controlled in vivo experimental study in rats using a whole-body γ-radiation–induced hepatotoxicity	Biochemical markers of oxidative stress and antioxidant defense, including hepatic malondialdehyde (MDA), reduced glutathione (GSH), superoxide	γ-radiation induced marked hepatic injury characterized by increased oxidative stress, elevated	the sample size per group was small, randomization and blinding procedures were not clearly described, and the	Positive (PRP alone).<br>Positive (PRP + low-molecular-weight chitosan

	gamma (γ)-radiation–induced hepatotoxicity (total dose 8 Gy, fractionated), with evaluation of PRP and/or low-molecular-weight chitosan as therapeutic interventions. PRO was obtained from 30 female rats	low-molecular-weight chitosan in ameliorating γ-radiation–induced hepatotoxicity in experimental rats, with emphasis on oxidative stress, antioxidant defense mechanisms, microRNA-21 regulation, and histopathological changes	model, including eight experimental groups (control, PRP alone, low-molecular-weight chitosan alone, PRP + chitosan, γ-irradiated, γ-irradiated + PRP, γ-irradiated + chitosan, and γ-irradiated + PRP + chitosan), with fractionated irradiation (total dose 8 Gy) and post-irradiation treatments, followed by biochemical, molecular, and histopathological assessments. Follow-up: 3 weeks	dismutase (SOD), and catalase activity; molecular assessment of hepatic microRNA-21 expression; histopathological evaluation of liver tissue architecture; and measurement of serum liver enzymes as indicators of γ-radiation–induced hepatotoxicity and therapeutic response to platelet-rich plasma and/or low-molecular-weight chitosan	liver enzymes, upregulation of microRNA-21, and pronounced histopathological alterations. Treatment with platelet-rich plasma significantly reduced oxidative damage, restored antioxidant enzyme activity, downregulated microRNA-21 expression, and improved liver histology. The combined treatment with PRP and low-molecular-weight chitosan produced greater protective and restorative effects than either treatment alone, indicating a synergistic therapeutic action against radiation-induced hepatotoxicity	study focused on an acute radiation injury model, which may limit extrapolation to chronic liver disease. PRP preparation was not standardized by platelet concentration, and functional hepatic outcomes beyond enzyme levels were not evaluated	showed stronger effect than PRP alone**)**
El-Sharouny et al. 2019 [34]	Adult male albino rats (n = 30, 10 rats/group) subjected to a cisplatin-induced hepatotoxicity experimental model	To analyze the therapeutic role of PRP in ameliorating cisplatin-induced hepatotoxicity in rats by evaluating biochemical liver enzymes,	A controlled in vivo experimental study in adult male albino rats using a cisplatin-induced hepatotoxicity model, including three groups (control, cisplatin-treated,	Serum biochemical markers of liver function (AST, ALT, ALP); hepatic oxidative and antioxidative stress markers, including malondialdehyde (MDA) and reduced glutathione (GSH); histological assessment of liver architecture (H&E staining);	Cisplatin administration induced marked hepatocellular injury characterized by elevated liver enzymes, increased oxidative stress, reduced antioxidant defenses, enhanced	The sample size was limited, PRP characterization was insufficiently detailed, and functional hepatic outcomes beyond enzyme levels were not assessed. The model reflects acute drug-induced	Positive (PRP decreased liver enzymes, lipidic peroxidation and improve the histologic architecture),

		oxidative and antioxidative markers, apoptotic activity, angiogenic response, and histopathological changes	and cisplatin + PRP), with PRP administered subcutaneously after cisplatin exposure and evaluation based on biochemical, oxidative/antioxidative, histopathological, histomorphometric, and immunohistochemical analyses. Follow-up: 4 weeks	quantification of collagen deposition (Masson's trichrome staining); histochemical evaluation of glycogen content (PAS reaction); immunohistochemical expression of apoptotic marker caspase-3 and angiogenic marker VEGF; and morphometric analysis of collagen area percentage and immunoexpression levels	apoptotic activity, and severe histopathological alterations. PRP treatment significantly ameliorated these effects by reducing liver enzyme concentrations, decreasing lipid peroxidation, restoring antioxidant enzyme activity, downregulating caspase-3 expression, enhancing VEGF expression, and improving hepatic histological architecture	hepatotoxicity, limiting extrapolation to chronic liver disease or fibrosis	
Aydın et al. 2019 [41]	Male Wistar albino rats (n = 34, 7-9 rats/group) experimental animals; additional 5 rats used for PRP preparation), subjected to a 70% partial hepatectomy–induced liver regeneration model	To investigate the effects of dexpanthenol, platelet-rich plasma, and thymoquinone on subacute liver regeneration after partial hepatectomy in rats by evaluating histopathological regeneration indices and oxidative stress–related biochemical parameters	A controlled in vivo experimental study in rats using a 70% partial hepatectomy model, including four groups (control, dexpanthenol (DX)-treated, PRP-treated, and thymoquinone (TQ)-treated), with daily intraperitoneal administration of the respective agents for 7 days, followed by histopathological and biochemical assessment of liver	Histopathological regeneration indices including ductus proliferation, inflammatory cell density in regenerated liver tissue, and inflammatory cell density in the regeneration zone; biochemical tissue markers of oxidative stress and antioxidant defense, including hydroxyproline, 8-isoprostane, 8-hydroxy-2'-deoxyguanosine, malondialdehyde, glutathione, and catalase levels	PRP significantly reduced all tissue oxidative stress and antioxidant-related biochemical parameters compared with control rats; however, it did not result in histopathological improvement of liver regeneration indices. DX significantly improved histopathological regeneration parameters without producing significant changes in	Short follow-up limited to the subacute regeneration phase, absence of molecular regeneration markers, small group sizes, and lack of combined treatment arms. PRP effects were confined to oxidative stress reduction without evidence of enhanced tissue regeneration	DX: positive histological effect with neutral biochemical impact<br>PRP: positive biochemical effect with neutral histological impact– TQ: overall neutral effect– No treatment achieved simultaneous histological and biochemical improvement

			regeneration at postoperative day 7. Follow-up: 1 week		biochemical oxidative stress markers. TQ showed no significant histopathological or biochemical effects on liver regeneration after partial hepatectomy		
Shoeib et al. 2018 [33]	Male albino rats; n = 80 (10 rats/group). Forty rats were used to prepare PRP, and the remaining forty rats underwent the experimental protocol (thioacetamide (TAA), hepatotoxicity/fibrosis model)	To evaluate the possible regenerative effect of PRP against TAA-induced hepatic damage	Controlled in vivo experiment in rats using a TAA-induced hepatic injury/fibrosis protocol, comparing vehicle controls and PRP controls versus TAA injury with/without PRP treatment (therapeutic administration starting during ongoing injury). Follow-up: 7 weeks	Biochemical liver function: serum ALT, AST, albumin. Oxidative/nitrosative stress: hepatic tissue peroxynitrite level; NAD(P)H quinone dehydrogenase 1 (NQO1) enzyme activity. Inflammation/fibrogenic signaling surrogates: hepatic tissue MIP-1α; hepatic tissue cAMP. Histopathology (H&E): hepatic architecture changes, inflammatory infiltrates, portal/pre-portal fibrosis features (qualitative microscopy)	TAA administration induced severe hepatic injury characterized by marked elevation of ALT and AST, reduced serum albumin, increased oxidative and nitrosative stress (elevated peroxynitrite), suppression of antioxidant defenses (reduced NQO1 activity), increased inflammatory signaling (elevated MIP-1α), decreased cAMP levels, and pronounced histopathological damage including inflammatory infiltration, fibrosis, and architectural distortion. PRP treatment significantly improved liver function tests, restored redox	absence of functional clinical outcomes, lack of fibrosis staging by standardized scoring systems, no long-term follow-up after PRP withdrawal, and limited translational relevance due to the exclusive use of a toxin-induced rat model	Positive (PRP produced concurrent biochemical, molecular, and histological improvement in TAA-induced liver injury, indicating a clear regenerative and antifibrotic effect

					balance, reduced inflammatory and fibrotic mediators, increased cAMP levels, and markedly improved hepatic histological architecture, with only minimal residual inflammatory infiltrates		
Salem et al. 2018 [32]	Adult male Wistar rats (n = 40, 10 rats/group) subjected to a dimethyl-nitrosamine (DMN)–induced liver fibrosis model	To investigate whether PRP can ameliorate biochemical, molecular, and histopathological alterations associated with DNM-induced liver fibrosis in rats and to elucidate the underlying antifibrotic, anti-inflammatory, and anti-apoptotic mechanisms	A controlled in vivo experimental study using a chemically induced liver fibrosis model, including four groups (control, PRP control, DMN-induced fibrosis, and DMN-induced fibrosis treated with PRP), with repeated PRP administration over three weeks and evaluation through biochemical assays, molecular gene expression analysis, and histopathological examination. Follow-up: 3 weeks	Serum liver enzymes (ALT, AST, GGT, LDH); hepatic hydroxyproline content as a marker of collagen deposition; inflammatory mediators (interleukin-8 and NF-κB1 expression); fibrotic markers (α-smooth muscle actin and TGF-β gene expression); anti-apoptotic marker (Bcl-2); and histopathological assessment of liver architecture	DNM administration induced severe liver injury characterized by marked elevations in liver enzymes, increased hepatic hydroxyproline and inflammatory markers, upregulation of profibrotic gene expression, reduced anti-apoptotic signaling, and pronounced histological fibrosis. Platelet-rich plasma treatment significantly improved liver enzyme profiles, reduced collagen deposition and inflammatory markers, downregulated fibrotic gene expression, increased Bcl-2 levels, and partially	Absence of functional liver outcomes beyond enzymes, lack of long-term follow-up, and reliance on a single PRP dosing regimen without platelet standardization	Positive (PRP improved liver enzymes, reduced fibrosis and inflammation markers, and promoted anti-apoptotic signaling

					restored normal hepatic architecture		
Fattah et al. 2018 [42]	Adult albino rats (male rats used for experimental groups), n = 80 males (10 rats/group), subjected to hepatotoxicity induced by lead nitrate, γ-radiation, or their combination. PRP was obtained from female rats.	To investigate the therapeutic effect of PRP against hepatotoxicity induced by lead nitrate and/or γ-radiation in rats and to explore the involvement of ERK1/2 and Akt signaling pathways in the protective mechanism	A controlled in vivo experimental study including eight groups: control, PRP control, lead nitrate, lead nitrate + PRP, γ-radiation, γ-radiation + PRP, lead nitrate + γ-radiation, and lead nitrate + γ-radiation + PRP. PRP was administered subcutaneously after toxic exposure, and outcomes were assessed using biochemical, oxidative stress, molecular signaling, and histopathological analyses. Follow-up: 5 weeks	Serum liver enzymes (ALT, AST), serum total protein and albumin; hepatic oxidative stress markers (MDA, nitric oxide); antioxidant defenses (GSH, GST, SOD, CAT); activation of ERK1/2 and Akt signaling pathways (phosphorylated and total protein levels); and histopathological evaluation of liver tissue architecture, using hematoxylin and eosin (H&E)–stained paraffin sections, with light microscopic assessment of hepatocellular degeneration, fibrosis, inflammatory infiltration, vascular congestion, and overall preservation or restoration of hepatic architecture	Lead nitrate and/or γ-radiation induced severe hepatotoxicity characterized by elevated liver enzymes, reduced protein synthesis, marked oxidative stress, and extensive histological damage. PRP treatment significantly improved liver function tests, restored antioxidant capacity, reduced oxidative damage, enhanced ERK1/2 and Akt activation, and markedly ameliorated histopathological liver injury across all toxicity models	Model complexity with multiple exposures, lack of fibrosis-specific quantification, and absence of long-term functional outcomes, which may limit direct translational interpretation	Positive (PRP restored liver architecture, reduced histopathological damage, and attenuated biochemical and oxidative stress alterations induced by lead nitrate and/or γ-radiation)
Mafi et al. 2016 [31]	Adult male Sprague–Dawley rats (n = 28, 7 rats/group) subjected to a CCl_4–induced hepatotoxicity and liver fibrosis	To investigate the therapeutic effects of PRP on liver structure and regeneration in a CCl_4-induced hepatotoxicity model using stereological and biochemical assessments	A controlled in vivo experimental study in rats using a chronic CCl_4-induced hepatotoxicity model, including four groups (control, CCl_4 alone, CCl_4 + PRP, and CCl_4 + saline), with a single PRP injection	Liver function tests (ALT, AST, ALP, albumin, total protein, bilirubin); stereological assessment of liver architecture including total liver volume, hepatocyte number, hepatocyte volume, sinusoidal space volume, and connective tissue volume using H&E and Masson's trichrome staining	PRP treatment significantly reduced connective tissue volume, indicating an antifibrotic effect. However, PRP also led to a significant reduction in hepatocyte number and volume, as well as decreased sinusoidal space	The study demonstrates a clear antifibrotic effect of PRP at the histological level but reveals concomitant negative effects on hepatocyte cellularity and architecture. Biochemical liver function was not improved, highlighting a	Negative (PRP had an antifibrotic effect but simultaneously decreased hepatocyte number and volume and did not improve liver enzyme levels, resulting in no overall functional or structural recovery)

	experimental model		administered via the anterior mesenteric vein and evaluation of biochemical liver markers and detailed stereological analysis of liver tissue. Follow-up: 11 weeks		volume. No significant improvement in serum liver enzymes or biochemical parameters was observed following PRP administration	dissociation between structural and functional outcomes and suggesting a dual, context-dependent effect of PRP	
Hesami et al. 2014 [43]	Male Wistar rats (n = 24, 6 rats/group) subjected to a CCl_4–induced chronic liver injury experimental model, with hepatotoxicity induced by repeated intraperitoneal CCl_4 administration over 8 weeks. 14 male rats were used for PRP preparation	To evaluate the hepatoprotective effects of PRP against chronic liver injury induced by carbon tetrachloride in rats	A controlled in vivo experimental study including four groups: control (olive oil), CCl_4-induced liver injury, PRP alone, and CCl_4-induced liver injury treated with PRP, with biochemical, oxidative stress, and histopathological assessment after chronic toxin exposure. Follow-up: 8 weeks	Serum liver enzymes (ALT, AST, albumin); hepatic oxidative stress markers including lipid peroxidation (TBARs) and reduced glutathione (GSH); and histopathological evaluation of liver architecture assessing necrosis, fatty change, ballooning degeneration, and inflammatory infiltration	CCl_4 induced marked hepatocellular injury characterized by elevated ALT and AST levels, increased lipid peroxidation, reduced glutathione depletion, and severe histopathological damage. PRP treatment significantly attenuated enzyme elevations, reduced oxidative stress, restored hepatic GSH levels, and markedly improved liver histology, showing reduced necrosis, fatty changes, and inflammation	Small sample size, lack of molecular pathway analysis, and absence of long-term functional outcomes beyond enzyme normalization	Positive (PRP attenuated CCl_4-induced liver injury by reducing histological damage, lowering serum ALT and AST levels, decreasing lipid peroxidation, and restoring hepatic glutathione levels, indicating both hepatoprotective and antioxidant effects)
Matsuo et al. 2011 [44]	Male Sprague–Dawley rats subjected to a 70% partial hepatectomy	To evaluate whether platelet-rich plasma administered via the portal vein enhances	A controlled in vivo experimental study in rats undergoing 70% hepatectomy, comparing portal	Liver regeneration assessed by liver-to-body weight ratio; hepatocyte proliferation evaluated by Ki-67 labeling index; activation of regenerative signaling	Portal vein infusion of PRP significantly increased liver-to-body weight ratio and hepatocyte	Short follow-up, absence of fibrosis or chronic liver injury models, and focus on acute regeneration	Positive (PRP infusion significantly increased liver regeneration after 70% hepatectomy and, increased

	model of liver regeneration, with portal vein infusion of PRP. n: not explicitly reported (at least 20 and up to ~50 rats, depending on overlap between cohorts)	liver regeneration after 70% hepatectomy and to characterize the associated regenerative signaling pathways	vein infusion of platelet-rich plasma versus normal saline immediately after surgery, with assessment of liver mass recovery, hepatocyte proliferation, platelet dynamics, liver function tests, histology, and intracellular signaling pathways. Follow-up: 24 h	pathways (Akt, ERK1/2, STAT3); platelet accumulation in hepatic sinusoids by intravital microscopy; serum liver function tests (ALT, AST, ALP, LDH, bilirubin); histological evaluation of liver injury	proliferation compared with controls. PRP induced earlier and sustained activation of Akt and ERK1/2 pathways, enhanced platelet accumulation in hepatic sinusoids, and promoted liver regeneration without inducing biochemical or histological liver damage	rather than long-term functional outcomes	hepatocyte proliferation (Ki-67)

Table S2. Methodological quality assessment of PRP preparation and characterization in included preclinical liver studies.

	Domain (1)								
**Authors and year**	**D1**	**D2**	**D3**	**D4**	**D5**	**D6**	**D7**	**Observations**	**Overall merit**
Chen et al. 2025 [38]	A%: citrate (10%), WBV: 10 mL B/rat, BVAR (10:1, TC: NR, CT: NR	MU: Manual double centrifugation method. CP: NR (RPM reported without rotor radius)	HT: top plasma extraction (1st centrifugate). Bottom plasma extraction (2nd centrifugate) . FPRPV: NR	BCC: NR. PLT-PRP: 3800 × 10^3 µL. LPRP: NR. PY: NR. AD: NR	SMQ: NR. ALA : 10% calcium chloride (CaCl2 (10:1))	NC: NR. PC NR. AC: PRP was physiologically activated	BDL-PRP group received an intraperitoneal (IP) injection of PRP in phosphate-buffered saline (PBS) twice a week for 4 weeks at a rate of 0.5 mL/kg after activation with calcium chloride.	PRP was diluted at 50% in PBS, thus the PRP dose of PLTs was of 1900 × 10^3 µL. There was not possible to stablish if the PRP used was autologous (AUT) or allogeneic (ALL)	Very low
Shivaramu et al. 2024 [37]	A%: NR. WBV: NR. BVAR: NR. TC: NR. CT: NR	MU: Manual double centrifugation method. CP: NR (RPM reported without rotor radius)	HT: NR. FPRPV: NR	BCC: NR. PLT-PRP: 307-705 × 10^3 µL. LPRP: NR. AD: NR	SMQ: NR. ALA: PRP was frozen (–°20)/thaw and then activated with CaCl2 (0.8 mL PRP + 0.2 mL 10% CaCl2)	NC: NR. PC: NR. AC: PRP was frozen (–°20)/thaw and then activated with CaCl2 (0.8 mL PRP + 0.2 mL 10% CaCl2)	PRP was administered in a bile duct ligation–induced model of cholestatic cirrhosis at a dose of 0.5 mL/kg, twice weekly from weeks 2 to 6.	The PRP source was ALL. Frozen PRP is a platelet lysate (PL), thus the addition of CaCl2 is unnecessary because PLTs were dead at the moment of application.	Very low

Bayoumi et al. 2023 [39]	A%: 3.8% sodium citrate (SC). WBV: 5-7 mL/rat. BVAR: NR. TC: NR. CT: °20	MU: Manual double centrifugation method. CP: NR (RPM reported without rotor radius)	HT: Plasma collected, supernatant discarded, platelet pellet resuspended. FPRPV: 1mL	BCC: NR. PLT-PRP: NR. LPRP: NR. AD: NR	SMQ: NR. ALA: NR	NC: NR. PC: NR. AC: NR	Heterologous (rat) PRP was administered IP (500 µL/kg, diluted 1:1 with PBS, administered twice weekly for 3 weeks), and intrahepatic (IH): single intrahepatic injection of 100 µL	PRP was diluted at 50% in PBS prior application	Very low
Elzaher et al. 2021 [36]	A%: SC. WBV: 2 mL/rat. BVAR: NR. TC: NR. CT: NR	MU: Manual double centrifugation method. CP: NR (RPM reported without rotor radius)	HT: plasma fractionation with removal of PPP and PAP; PRP collected from bottom plasma layer. FPRPV: NR	BCC: NR. PLT-PRP: NR. LPRP: 900-1000 × 10^3 µL. AD: NR	SMQ: NR. ALA: NR	NC: NR. PC: NR. AC: NR	AUT PRP was administered in a carbon tetrachloride–induced model of chronic liver fibrosis at a dose of 1 mL/kg, twice weekly for four consecutive weeks following fibrosis induction	The authors did not report whether PRP was activated prior to administration, nor did they provide details regarding activation agents or protocols	Very low
Almaali et al. 2021 [40]	A%: sodium citrate (SC). WBV: NR. BVAR: 9:1. TC: NR. CT: NR.	MU: Manual centrifugation method. CP: 1st C: 4000 × g /10 min. 2nd C: 800 × g / 10 min	HT: upper two/third layer discarded. FPRPV: 0.3 mL/rat.	BCC: NR. PLT-PRP: NR. LPRP: NR. PY: NR. AD: NR.	SMQ: NR. ALA: NR.	NC: NR. PC: NR. AC: NR.	ALL PRP was administered in a diclofenac-induced model of combined hepatic and renal toxicity as a single subcutaneous dose of 0.3 mL per rat, given at the beginning of the experiment, followed by daily intraperitoneal diclofenac administration for 28 days.	The authors did not report whether PRP was activated prior to administration, nor did they provide details regarding	Very low

								activation agents or protocols	
Saif-Elnasr et al. 2019 [35]	A%: 3.8%. WBV: NR. BVAR: 9:1. TC: NR. CT: 20 °C.	MU: Manual double centrifugation protocol. CP: NR (RPM reported without rotor radius)	HT: erythrocytes discarded; PPP aspirated; platelet pellet resuspended. FPRPV: 1 mL (PLT pellet resuspended in 1mL of platelet-poor plasma (PPP))	BCC: NR. PLT-PRP: ~5× WB concentration. LPRP: NR. PY: NR. AD: hemocytometer	SMQ: NR (no GF/cytokine quantification in PRP). ALA: $CaCl_2$ 10%, 50 μL per 1 mL PRP; incubated 1 h at 37 °C	NC: NR. PC: NR. AC: PRP activated with $CaCl_2$ after preparation (supernatant collected after centrifugation)	PRP supernatant was administered in a γ-radiation–induced hepatotoxicity model at a dose of 0.5 mL/kg, subcutaneously, twice weekly during irradiation and for one additional week after the last radiation dose	True PRP was not used. The authors only used a part of PRP that is its supernatant. The values for BCCs were not reported, thus the PLT concentration in PRP is uncertain	Very low
El-Sharouny et al. 2019 [34]	A%: sodium citrate (3.8%). TBV: NR. BVAR: 9:1. TC: NR. CT: 20 °C	MU: manual double centrifugation. CP: NR (RPM reported without rotor radius)	HT: platelet pellet resuspended in PBS after plasma separation. FPRPV: NR.	BCC: NR. PLT-PRP (679 × 10^3/μL). LPRP: NR. AD: Sysmex KX-21 hematology analyzer (Sysmex Corporation, Kobe, Japan)	SMQ: NR. ALA: PRP activated using calcium gluconate and thrombin with 1 h incubation	NC: NR. PC: NR. AC: NR	PRP filtered supernatant was administered in a cisplatin-induced acute hepatotoxicity model, following a single IP cisplatin dose (7.5 mg/kg) and subsequent subcutaneous PRP administration at 0.5 mL/kg, twice weekly for three weeks starting on day 7 after injury induction	True PRP was not used. The authors only used a part of PRP that is its supernatant. The values for BCCs were not reported. PRP source (either AUT or ALL) not reported	Very low
Aydın et al. 2019 [41]	A%: sodium citrate (3.2%). TBV: 5–6 mL/rat. BVAR:	MU: manual double-spin centrifugation. CP: 1st C 400 × g/10 min and	HT: two-thirds of the supernatant discarded after second	BCC: NR. PLT-PRP: NR. LPRP: NR. PY: NR. AD: NR	SMQ: NR. ALA: NR	NC: NR. PC: NR. AC: NR.	ALL PRP was administered in a 70% partial hepatectomy model at a dose of 1	It was not possible to stablish if PRP was used in this study.	Very low

	NR. TC: NR. CT: NR.	2nd C 800 × g/10 min; CT: NR.	centrifugation; remaining fraction accepted as PRP. FPRPV: NR.				mL/kg, IP, once daily for 7 consecutive days		
Shoeib et al. 2018 [33]	A%: sodium citrate (3.8%). TBV: NR. BVAR: 9:1. TC: NR. CT: 20 °C	MU: manual double-spin centrifugation. CP: NR (RPM reported without rotor radius)	HT: PRP obtained after removal of PPP and resuspension of platelet pellet in PBS. FPRPV: NR	BCC: platelet count reported in PRP (range approximately 800–1700 × $10^3/\mu L$), LPRP: NR. PY: NR. AD: NR.	SMQ: NR. ALA: NR	NC: NR. PC: NR. AC: NR.	ALL PRP was administered in a thioacetamide-induced model of chronic liver injury at a dose of 0.5 mL/kg, IP, twice weekly for four weeks following fibrosis induction	The authors did not report whether PRP was activated prior to administration, nor did they provide details regarding activation agents or protocols	Very low
Salem et al. 2018 [32]	A%: sodium citrate (3.2%). TBV: 2 mL per donor rat. BVAR: 2 mL blood / 0.3 mL anticoagulant. TC: NR. CT: NR.	MU: manual double-spin centrifugation. CP: NR (RPM reported without rotor radius)	HT: plasma fraction collected above the buffy coat, PPP discarded after second centrifugation, and platelet button resuspended in PBS at a 1:1 ratio. FPRPV: NR	BCC: NR. PLT-PRP: 1000 × $10^3/\mu L$ PY: NR. AD: MICROS abc LC-152 (Horiba Ltd., Kyoto, Japan)	SMQ: NR. ALA: PRP activated immediately before use with $CaCl_2$ (0.8 mL PRP + 0.2 mL 10% $CaCl_2$). Supernatants were frozen at −80 °C	NC: NR. PC: NR. AC: NR.	ALL PRP was administered in a dimethylnitrosamine-induced model of liver fibrosis at a dose of 0.5 mL per rat, delivered subcutaneously, twice weekly for three weeks	True PRP was not used. The authors only used a part of PRP that is its supernatant	Very Low
Fattah et al. 2018 [42]	A%: sodium citrate (3.8%). TBV: pooled blood from donor rats (individual volume NR). BVAR: 9:1. TC: NR. CT: room temperature	MU: manual double-spin centrifugation. CP: NR (RPM reported without rotor radius)	HT: PPP aspirated after second centrifugation and platelet pellet resuspended in 1 mL plasma to obtain PRP. FPRPV: 1 mL	BCC: NR. PL-PRP: NR (~ 5× tah in WB) LPRP: NR. PY: NR. AD: hemocytometer	SMQ: NR. ALA: PRP activated with $CaCl_2$ 10% at a ratio of 50 μL per 1 mL PRP and incubated for 1 h at 37 °C	NC: NR. PC: NR. AC: 10% CaCl2 activator solution was added to the PRP solution in a ratio of 50 μl CaCl2 for every 1 ml of PRP. The activated PRP was centrifuged at 4000 rpm and 4	ALL PRP supernatant was administered in lead nitrate– and/or γ-radiation–induced hepatotoxicity models at a dose of 0.5 mL/kg, subcutaneously, starting 24 h after the last toxic insult and continued twice weekly for three consecutive weeks	True PRP was not used. The authors only used a part of PRP that is its filtered supernatant	Very low

						°C for 10 min, then the supernatant was collected and stored in aliquots at−20 °C for subsequent use			
Mafi et al. 2016 [31]	A%: sodium citrate (3.2%). TBV: pooled blood from 7 donor rats (individual volume NR). BVAR: 9:1. TC: NR. CT: NR	MU: manual double-spin centrifugation. CP: 1st C 400 × g/10 min and 2nd C 800 × g/10 min	HT: upper two-thirds of the supernatant aspirated after the second centrifugation and the remaining layer considered as PRP. FPRPV: NR	BCC: 603 × 10^3 PLT /μL. PLT-PRP: 1806 × 10^3/μL), indicating approximately threefold enrichment; LPRP: NR. PY: NR. AD: Sysmex XT-1600i (Sysmex Corporation, Kobe, Japan)	SMQ: NR. ALA: NR (PRP frozen at −20 °C prior to use, no activation protocol described)	NC: NR. PC: NR. AC: NR	ALL PRP supernatants were administered in a carbon tetrachloride–induced hepatotoxicity model as a single intra-mesenteric vein injection of 1 mL, delivered 24 h after the last CCl_4 administration, with animals sacrificed one week after PRP treatment	True PRP was not used. The authors only used a part of PRP that is its filtered supernatant	Very low
Hesami et al. 2014 [43]	A%: sodium citrate (3.8%). TBV: pooled blood (100 mL) obtained. BVAR: 9:1. TC: NR. CT: 20 °C	MU: manual double-spin centrifugation. CP: NR (RPM reported without rotor radius)	HT: PPP removed after second centrifugation and platelet pellet resuspended in PBS; platelet concentrate was pooled and incubated at room temperature to reduce aggregation. FPRPV: NR	BCC: NR. PRP-PRP: 679 × 10^3/μL. LPRP: NR. PY: NR. AD: Sysmex KX-21 (Sysmex Corporation, Kobe, Japan)	SMQ: NR (only reported total protein). ALA: PRP activated using autologous thrombin prepared with calcium gluconate (100 mg/mL), incubated for 1 h at room temperature, followed by centrifugation and filtration (0.22 μm); supernatant stored at −80 °C	NC: NR. PC: NR. AC: platelet releasate was obtained after activation and removal of platelet membranes by centrifugation	ALL PRP supernatant was administered in a CCl_4-induced chronic liver injury model at a dose of 0.5 mL/kg (1:1 in PBS), subcutaneously, twice weekly for three weeks, starting five weeks after initiation of CCl_4 exposure	True PRP was not used. The authors only used a part of PRP that is its filtered supernatant	Very low
Matsuo et al. 2011 [44]	A%: acid citrate dextrose (ACD). TBV: NR. BVAR: 1:4.	MU: manual centrifugation protocol with preparation of	HT: PRP further processed to obtain washed	BCC: NR. PLT-PRP: 1000 × 10^3 platelets/μL.	SMQ: NR. ALA: NR (no activation protocol	NC: NR. PC: NR. AC: NR	Washed ALL PRP was administered in a 70% partial hepatectomy model as a	The intervention corresponds to washed platelet	Very low

	TC: NR. CT: 24 °C (first centrifugation) and 4 °C (washing steps)	washed platelets. CP: 1st C 200 × g/10 min/24 °C followed by washing centrifugations at 1000 × g/15 min/4 °C	platelets (PRP), which were resuspended in citrate buffer and finally in normal saline. FPRPV: adjusted to a final concentration of 1000 × 10^3 platelets/μL	LPRP: NR. PY: NR. AD: NR	described; platelets administered as washed, non-activated suspension		single portal vein infusion of 1 mL immediately after hepatectomy, corresponding to approximately 10% of the total circulating platelet count, with animals followed for up to 24 h for liver regeneration and early signaling outcomes	infusion rather than conventional PRP, limiting direct comparability with later PRP-based studies	

Domain 1 (Blood Collection & Pre-processing), the following parameters are assessed: A% (Anticoagulant type and concentration); WBV (Whole blood volume); BVAR (Blood-to-anticoagulant ratio); TC (Time to centrifugation); and CT (Centrifugation temperature). Domain 2 (PRP Preparation) uses: MU (Method used); and CP (Centrifugation parameters). Domain 3 (PRP Harvesting) uses: HT (Harvesting technique); and FPRPV (Final PRP volume). Domain 4 (Cellular Characterization) uses: BCC (Basal cell counts); PLT-PRP (Platelet concentration in PRP); LPRP (Leukocyte concentration in PRP); PY (Platelet yield); and AD (Analytical device). Domain 5 (Biochemical Characterization) uses: SMQ (Soluble mediator quantification); and ALA (Activation or lysis method for assay). Domain 6 (Protein Enrichment Controls) uses: NC (Negative control); PC (Positive control); and AC (Activation clarification). NR (Not reported); PBS (Phosphate-buffered saline); PPP (Platelet-poor plasma); IP (Intraperitoneal); IH (Intrahepatic); SC (Subcutaneous); AUT (Autologous); ALL (Allogeneic); PL (Platelet lysate); and $CaCl_2$ (Calcium chloride)
